# Virtual screening using covalent docking to find activators for G245S mutant p53

**DOI:** 10.1371/journal.pone.0200769

**Published:** 2018-09-07

**Authors:** Sara Ibrahim Omar, Marco Gaetano Lepre, Umberto Morbiducci, Marco Agostino Deriu, Jack A. Tuszynski

**Affiliations:** 1 Department of Oncology, Faculty of Medicine and Dentistry, University of Alberta, Edmonton, Alberta, Canada; 2 Department of Mechanical and Aerospace Engineering, Politecnico di Torino, Torino, Italy; 3 Istituto Dalle Molle di studi sull’Intelligenza Artificiale (IDSIA), Scuola universitaria professionale della Svizzera italiana (SUPSI), Università della Svizzera italiana (USI), Centro Galleria 2, Manno, Switzerland; 4 Department of Physics, Faculty of Science, University of Alberta, Edmonton, Alberta, Canada; Virginia Commonwealth University, UNITED STATES

## Abstract

*TP53* is the most mutated gene in all cancers. The mutant protein also accumulates in cells. The high frequency of p53 mutations makes the protein a promising target for anti-cancer therapy. Only a few molecules have been found, using *in vitro* screening, to reactivate the mutant protein. APR-246 is currently the most successful mutant p53 activator, which reactivates the transcriptional activity of p53 by covalently binding to C124 of the protein. We have recently created *in silico* models of G245S-mp53 in its apo and DNA-bound forms. In this paper we further report on our *in silico* screening for potential activators of G245S-mp53. We filtered the ZINC15 database (13 million compounds) to only include drug-like molecules with moderate to standard reactivity. Our filtered database of 130,000 compounds was screened using the DOCKTITE protocol in the Molecular Operating Environment software. We performed covalent docking at C124 of G245S-mp53 to identify potential activators of the mutant protein. The docked compounds were ranked using a consensus scoring approach. We also used ADMET Predictor™ to predict pharmacokinetics and the possible toxicities of the compounds. Our screening procedure has identified compounds, mostly thiosemicarbazones and halo-carbonyls, with the best potential as G245S-mp53 activators, which are described in this work. Based on its binding scores and ADMET risk score, compound 2 is likely to have the best potential as a G245S-mp53 activator compared to the other top hits.

## Introduction

The transcription factor, p53, binds to its response elements to activate the transcription of canonical p53 target genes[1]. It controls various processes in cells such as DNA repair, cell proliferation, metabolism, senescence and apoptosis[1–3]. Since p53 is a master tumor suppressor protein, it is not surprising that it is the most mutated protein in all cancer types[4]. Mutations in the *TP53* gene often result in a p53 mutant protein that loses its specific DNA binding ability, which consequently compromises or abolishes the protein’s tumor suppression function[5,6]. The great importance of p53 in the context of cancer has made it a logical target for anti-cancer treatment[2]. Indeed, *in vivo* studies have shown that reconstitution of the wild type (wt) p53 activity in mice induces rapid tumor regression even in the presence of other tumor-associated genetic alterations[7,8].

A synthetic nine amino acid peptide CDB3 (REDEDEIEW) has been found in a peptide screen to stabilize the p53 core domain[9]. Additionally, it was found to restore the sequence-specific binding of the I195T mp53 to DNA[9]. CDB3 was derived from the p53 binding protein, apoptosis-stimulating of p53 protein 2 (ASPP2), which enhances the DNA binding and transactivation ability of p53 and consequently enhances the apoptotic function of the latter protein[10]. It has been hypothesized that CDB3 acts as a chaperone that binds to p53 and shifts the equilibrium of the protein folding towards its native state. CDB3 is then displaced by the specific DNA sequence[9]. Such chaperone peptides have some obvious limitations due to their large size and poor bioavailability. In addition, they are unlikely to restore the DNA binding ability of contact mutants of p53.

Small molecule p53 activators, rather than peptides, have more advantages as therapeutic agents in terms of bioavailability, drug administration and compound synthesis. Attempts to develop small molecules aimed at restoring the wt activity to mutant p53 (mp53) have progressed in the past years. In 1999, Foster and colleagues[11] reported the discovery of CP-31398, obtained by a library screen based on an *in vitro* biochemical assay, in which antibodies were used to distinguish between the wt and mutant conformations of p53. CP-31398 was also shown to have a stabilizing effect on the p53 DNA binding domain (DBD) and enhance the transcriptional activity of wt-p53 in tumor xenografts expressing the mutant protein[11,12]. However, determination of a detailed mechanism of action of CP-31398 still remains elusive[13]. A study has shown that the molecule binds tightly to the DNA[14] and another suggested that the molecule acts on other targets since CP-31398 altered gene expression in both p53 dependent and independent manners[15].

Other successful attempts at finding mp53 rescuers have identified PRIMA-1 (‘p53 reactivation and induction of massive apoptosis’) and MIRA-1 by means of an *in vitro* screen[16,17]. The methylated derivative of PRIMA-1, called APR-246[18], is the only small molecule mp53 activator that has reached clinical trials[19]. Both PRIMA-1 and APR-246 are prodrugs that decompose into the methylene quinuclidinone (MQ). The active MQ, characterized by a reactive double bond, was found to react with the cysteine residues of p53 through a Michael addition reaction, which restores the wt conformation and transcriptional activity of the protein[18]. *In silico* analysis using molecular dynamics (MD) identified a transiently open pocket in the DBD of p53 formed between loop L1 and the S3 beta-sheet, which contains three cysteines at residues 124, 141 and 135[20]. In that study, the wt, R175H, R273H and G245S mp53 proteins were simulated. The calculated solvent-accessible surface area of the three different cysteines revealed that C124 was the most solvent exposed cysteine residue at that pocket. Hence, C124 was concluded to be the most likely residue at which MQ reacts with mp53 to restore its wt transcriptional activity[20]. This conclusion was further confirmed by the results from the site-directed mutagenesis of C124 to alanine[20]. MQ treatment could not inhibit the growth of Saos-2 cells in C124A-R175H-mp53 vs. R175H-mp53 transfected cells. Wassman *et al*. further performed virtual screening using non-covalent docking of the NCI diversity set II and identified stictic acid as a novel mp53 activator. Indeed, stictic acid was found to elicit the activation of p21, a p53 target gene product, in a dose-dependent manner in Saos-2 cells transfected with R175H-mp53. Additionally, stictic acid and MQ increased the thermal stability of R175H and G245S mp53.

We have previously non-covalently docked small molecule activators of mp53 to R273H-mp53[21]. The docked covalent p53 activators included MQ, NB, STIMA-1, MIRA-1 and CP-31398[21]. While the five compounds were not predicted to interact directly with C124, they were within a short distance that would allow the reaction of the double bonds of the molecules with the thiol group of C124. On the other hand, our docking results of the non-covalent p53 activators showed that the activator molecules interact directly with C124. In the same study, we also used ADMET Predictor to predict the pharmacokinetics and toxicity of the docked compounds. Although *in silico* toxicity predictions indicated that stictic acid is less toxic than MQ, the former compound was predicted to have poor pharmacokinetic properties.

The highest frequency, hotspot, mutations in p53 are categorized based on how the mutations alter the protein’s binding to the DNA. p53 variants with a mutation in a residue that natively interacts with the DNA in the wt protein are classified as contact mutants. Other mutations in the DNA binding domain of p53 are classified as structural mutants, since they alter the protein’s structure and therefore affect its binding to the DNA[22]. G245S-mp53 is one of the p53 hotspot structural mutants, which carries a single-point mutation in codon 245 that changes the wild-type glycine residue to serine[22].

As explained above, previous efforts at finding p53 activators were mainly based on *in vitro* and *in vivo* studies[9,13,18]. *In silico* screening at C124 has been used to find stictic acid[20]. Docking at a cleft near loop L6 was also used to screen a library of two million compounds to find activators of Y220C-mp53, which successfully yielded PhiKan083 using non-covalent docking[23]. In this study, we used DOCKTITE[24], a covalent docking protocol, to screen a subset of the ZINC database at the C124 pocket to find potential activators of G245S-mp53. To refine our predictions, we also used a consensus scoring approach by combining two scoring functions to improve the pose and binding energy predictions. Here, we report potential G245S-mp53 activators and some of their predicted ADMET properties in this work.

## Results and discussion

### G245S-mp53 protein models

We performed MD simulations of the apo G245S-mp53 as well as the DNA bound protein obtained from the virtual mutation of G245 of wt-p53 to serine in the experimentally determined structures with PDB ID: 2FEJ and 4HJE, respectively. This was to account for the different possible conformations of G245S-mp53. We also used the representative structures from the MD simulations to account for the protein’s flexibility specifically at the binding site. These representative structures were obtained by clustering the equilibrated protein[25] based on the RMSD of residues 113–124 and 141–146, which constitute the pocket around the reactive C124 residue. Clustering was performed based on the average-linkage algorithm[26] using the cpptraj utility in Ambertools[27]. The choice of the optimum number of clusters was guided by the calculated DBI, pSF and SSR/SST clustering metrics for each cluster count from 2 to 20.

**Fig 1:**
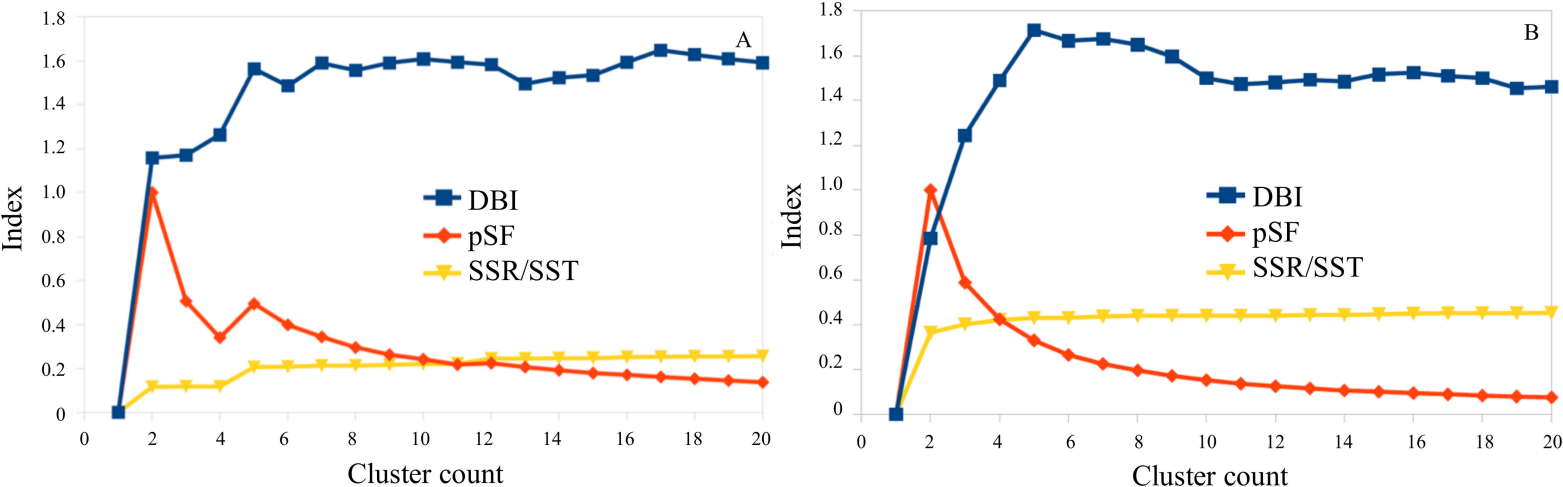
Plots of the DBI, pSF and SSR/SST clustering metrics for the equilibrated (A) apo and (B) DNA-bound G245S-mp53. The pSF values were normalized to fit on the graph.

The choice of the optimum cluster number is not trivial. Ideally, the best number of clusters falls at a local minimum DBI value, local maximum pSF value and where the SSR/SST ratio starts to plateau[26]. Our clustering metrics did not all fulfill these criteria at a particular cluster count, as shown in Fig 1. As partial fulfilment of these criteria, however, we chose cluster counts of 4 and 2 to represent the last 460 ns and 1 μs of the equilibrated apo and DNA-bound G245S-mp53. The centroids of these clusters were used to represent the G245S-mp53 for covalent docking. We used monomer B in the DNA-bound G245S-mp53 models and removed the bound DNA for docking.

### Covalent docking of the filtered ZINC library

While non-covalent docking is a vastly used virtual screening tool, covalent docking has been used less frequently in computational drug design. Nonetheless, covalent docking has been used successfully[28–30]. A previous study employing virtual screening using covalent docking on the vaccinia virus I7L ubiquitin-like proteinase homology model has reported a 21% successful hit rate[31].

In this study, we used a versatile covalent docking protocol in the Molecular Operating Environment (MOE)[32] called DOCKTITE[24]. We assigned the thiol of C124 as the reaction site for the screened library. We filtered the ZINC library to only include compounds that were in stock and had a molecular weight between 300 and 500, which falls within the weight range suggested by the Lipinski’s rule of five[33]. We also filtered for compounds that had moderate to standard reactivity. This criterion was specified since we aimed to find covalent activators of p53 that could permanently restore the mutant protein’s wild type activity. The final filtered library size was about 130,000 molecules.

Each of the screened ligands was then tagged at its reaction site and additional stereoisomers were created for ligands with prochiral centers. A conformational search was performed in MOE until a maximum of 5,000 conformers were generated for each ligand and each isomer. A pharmacophore model was automatically generated by MOE to guide the placement of the generated conformers at the active site. The docked poses were then evaluated by the Affinity dG scoring function in MOE. The top 100 poses of each docked ligand were further refined. There are two possible refinement methods in MOE: energy and grid minimization. ROC curves of the two methods have demonstrated that the former is only marginally more accurate with an area under the curve of 0.81 vs. 0.79[24]. This slight increase in accuracy comes at the expense of forty times the computational cost[24]. For our virtual screening purposes we, therefore, used grid minimization for refinement and rescored the refined poses using the Affinity dG scoring function.

**Fig 2:**
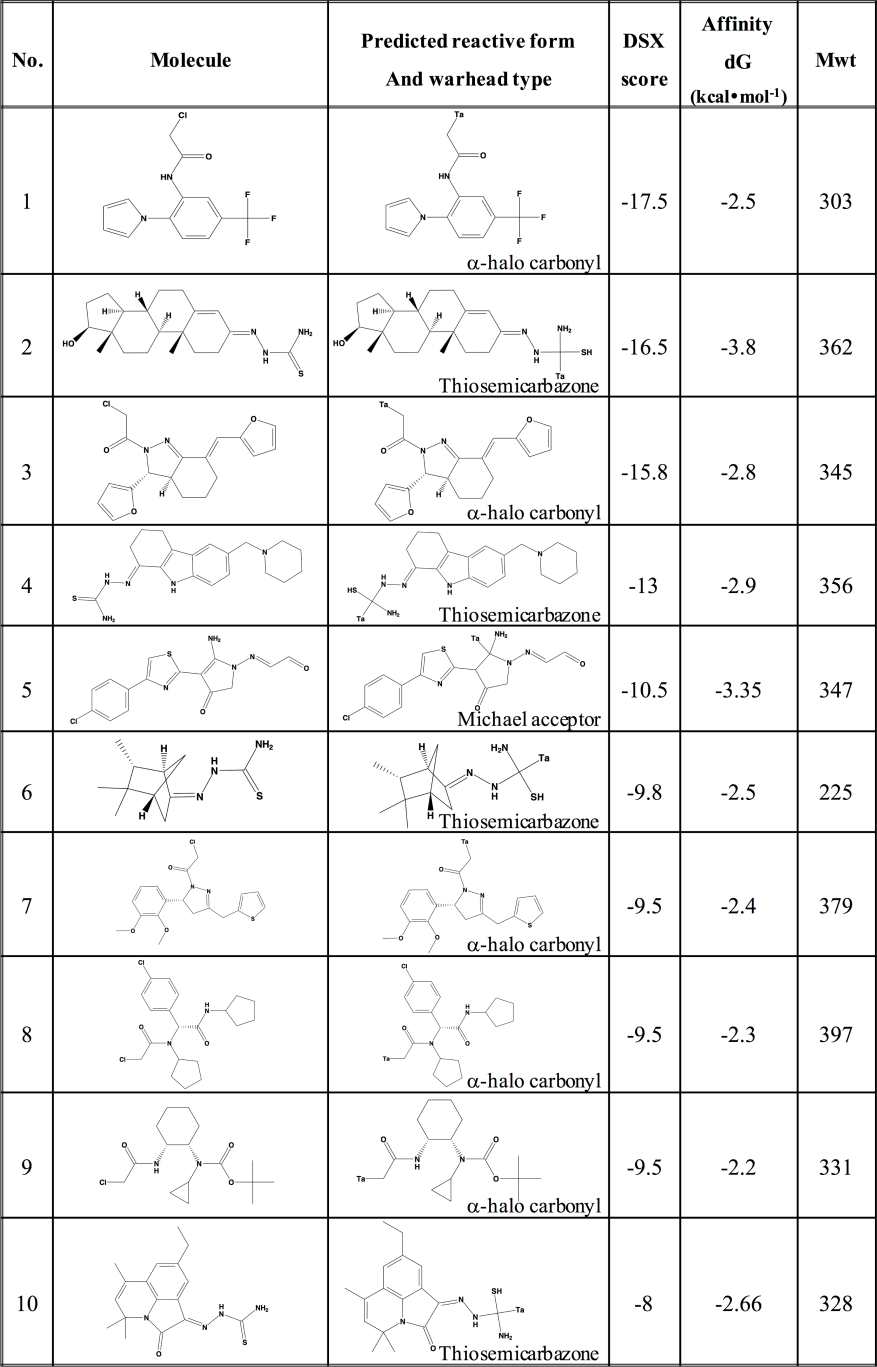
The top ten hits from our covalent docking virtual screening. The hits are ranked based on their DSX score. The reactive moiety of molecules is tagged by ‘Ta’, which marks the thiol group of C124 of G245S-mp53 in case of a true hit. Mwt = molecular weight of the compound.

We used a consensus-based strategy to rank the top hits. To do this, the highest ranked pose for each compound was then detached and rescored by the DSX scoring function as a non-covalently bound ligand since it gives more accurate results[24]. While the Affinity dG scoring function predicts the binding energy of the ligand, the DSX is a knowledge-based function that scores ligands on how close they are to near-native poses i.e. experimentally resolved complex structures[24]. The screened compounds were ranked based on both scores. Fig 2 shows a list of the top 10 potential activators based on their DSX scores and favorable predicted Affinity dG scores.

Unlike all the previous covalent p53 activators such as MQ, STIMA-1 and MIRA-1, which are all Michael acceptors, five of our potential hits were thiosemicarbazones, four were halo-carbonyls and only one was a Michael acceptor molecule (Fig 2). The DSX scores, which rank near-native binding modes, ranged from -17.5 to -8. The Affinity dG binding energies of the compounds ranged from -3.8 to -2.2 kcal•mol^-1^.

In conventional non-covalent docking, especially when screening for competitive inhibitors, a higher binding affinity is an indication of better potential inhibition. This is due to the fact that a ligand that can bind strongly at the active-site will likely decrease the chance of the native substrate from binding. However, analysing the results of covalent molecules aimed at activating a protein, like our case, is less trivial. A good p53 mutant activator is a compound that not only interacts with C124, like MQ, but also alters the structure of the protein to restore its wild type activity. Nonetheless, a higher DSX score, being an indication of the pose being near-native, is an indication that the conformation is likely to exist. Indeed, a 21% hit rate was achieved in a previous study where virtual screening using covalent docking was used.

### ADMET property predictions

The toxicities of the top 10 compounds were predicted using ADMET Predictor™ and were compared to those of APR-246’s active metabolite called MQ. The prodrug APR-246 is the only mp53 activator that is currently in clinical trials[19]. Some of the predicted properties are listed in Table 1.

**Table 1:** The predicted ADMET properties of MQ and the top 10 potential hits from our covalent docking screen. Values between brackets indicate confidence levels.

Compound	ADMET risk score	BBB filter	hERG filter	Pgp Inh	Pgp substr	Ser ALT	Ser AST
1	3.8	High	Yes (85%)	No (94%)	No (95%)	Elevated (98%)	Elevated (94%)
2	1.0	High	No (95%)	No (63%)	No (59%)	Normal (78%)	Normal (62%)
3	2.9	High	No (95%)	Yes (83%)	No (95%)	Normal (73%)	Elevated (94%)
4	3.7	High	No (95%)	Yes (69%)	Yes (97%)	Normal (52%)	Normal (70%)
5	6.0	High	No (59%)	No (65%)	No (58%)	Elevated (84%)	Elevated (94%)
6	2.8	High	No (95%)	No (94%)	No (85%)	Normal (95%)	Normal (65%)
7	0.4	High	No (95%)	No (65%)	No (95%)	Normal (60%)	Elevated (69%)
8	4.4	High	No (82%)	Yes (70%)	No (95%)	Elevated (66%)	Elevated (94%)
9	1.0	High	No (76%)	No (62%)	No (79%)	Normal (99%)	Normal (89%)
10	4.2	Low	No (95%)	No (94%)	No (59%)	Elevated (70%)	Elevated (59%)
MQ	3.0	High	No (95%)	No (94%)	No (75%)	Elevated (79%)	Elevated (94%)

ADMET Predictor™ assigns an ADMET risk score to each compound based on its calculated pharmacokinetic (PK) and pharmacodynamics (PD) properties; higher scores are assigned to less favorable properties. This parameter has been developed using drugs from the World Drug Index as a training set. Only 10% of the focused subset of the World Drug Index have an ADMET risk score of more than 6.5. MQ had a predicted risk score of 3. Only compounds 2, 6, 7 and 9 had lower risk scores than MQ (Table 1). Interestingly, all the top hits as well as MQ, are predicted to cross the blood brain barrier, except compound 10. While compound 1 was predicted to be cardiotoxic, the remaining compounds, including MQ, were not predicted to inhibit the hERG channel. Additionally, most compounds were predicted to be non-inhibitory to p-glycoproteins, nor were substrates to these protein pumps. The only exceptions to this were compounds 3 and 8, which were predicted to inhibit p-glycoproteins. Compound 4 was predicted to be both a p-glycoprotein substrate and inhibitor. Like MQ, compounds 1, 5, 8 and 10 were predicted to be hepatotoxic and likely to cause an elevation in serum levels of both ALT and AST. None of the other compounds are expected to elevate serum levels of the two enzymes, except compounds 3 and 7, which were predicted to elevate serum AST levels. It is worth noting that none of our potential hits violates Lipinski’s rule of five[33].

### Compound 2: The best potential hit

From the results above, compound 1 had a DSX score of -17.5, an Affinity dG score of -2.5 kcal•mol^-1^ and an ADMET risk score of 3.8. Compound 2, which had the second highest DSX score of -16.5, had the highest Affinity dG score of -3.5 kcal•mol^-1^ and the second best ADMET score of 1. The compound with the best ADMET risk score of 0.4, had DSX and Affinity dG scores of -9.5 and -2.4 kcal•mol^-1^, respectively. Collectively, these predictions indicate that compound 2 has the best potential as a G245S-mp53 activator.

**Fig 3:**
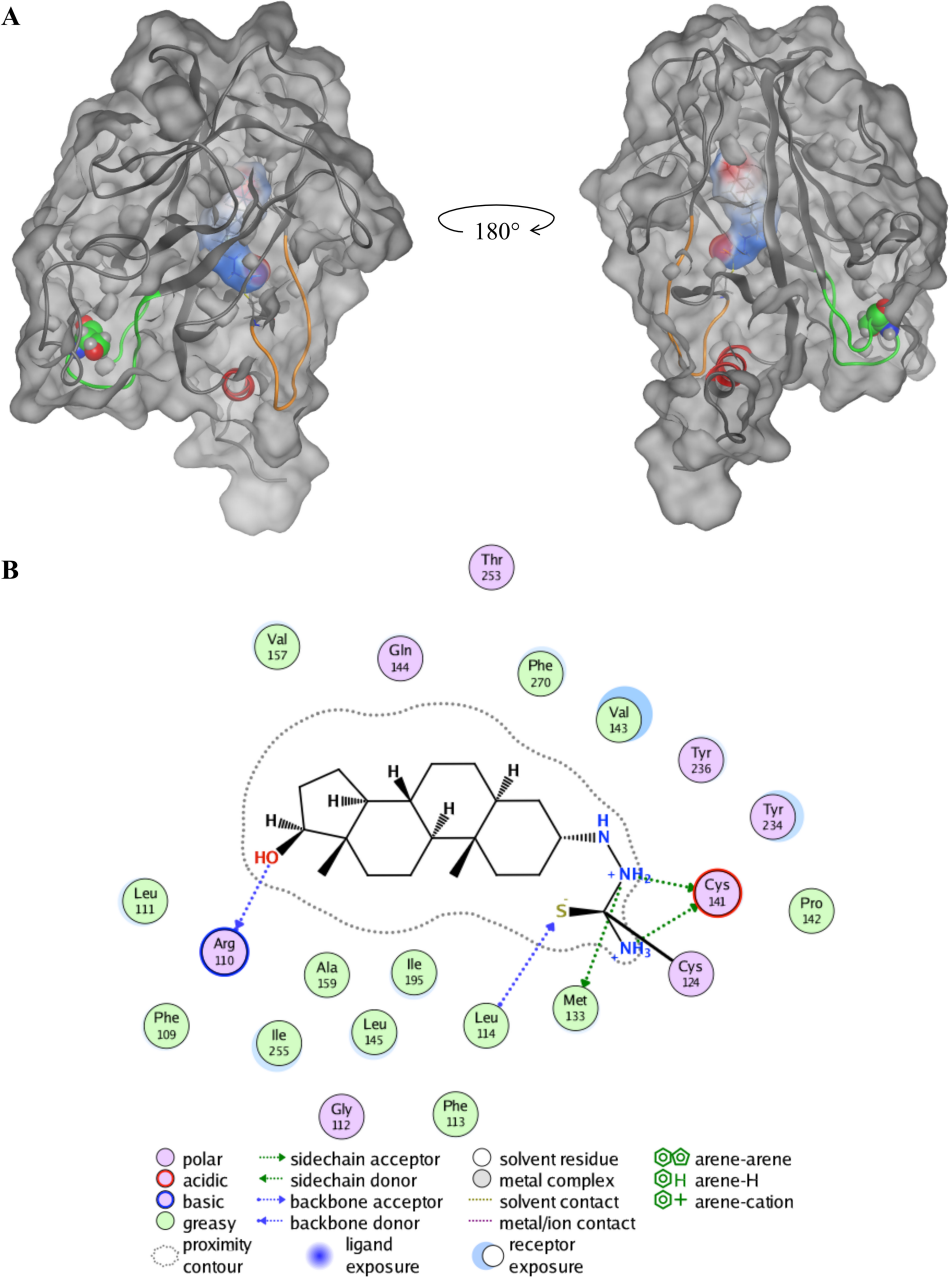
Compound 2 covalently bound to G245S-mp53. (A) The protein’s molecular surface is shown in grey. Loop L1, where compound 2 is covalently bound, is colored as an orange ribbon. Helix H2 and loop L3, which interact with the DNA are colored red and green, respectively. The mutated S245 in loop L3 is shown as green spheres. Compound 2 is represented by its molecular surface and is colored based on its electrostatic potential. (B) The ligand interaction scheme of compound 2 with the minimized G245S-mp53.

Fig 3 shows the minimized G245S-mp53 bound to compound 2 at C124 (S1 Text). Our model shows that compound 2 becomes buried in the core of the protein (Fig 3A). This is especially true for the fused ring system of compound 2, which becomes surrounded by F109, L111, F113, V143, L145, A159, I195, Y234, Y236, I255 and F270 (Fig 3B). Additionally, the amine group of compound 2 is predicted to be protonated while the neighboring C141 thiol of G245S-mp53 becomes deprotonated. These moieties are predicted to form a salt bridge. The amine group of compound 2 also forms a hydrogen bond with the backbone of C141 of the protein. Also, R110 backbone forms a hydrogen bond with the hydroxyl of compound 2. These interactions could likely confer a conformational change in G245S-mp53 that could lead to better binding of the protein to its response elements and hence restore the wild activity to this mutant.

## Conclusions

Restoring the wt activity to mp53 is a promising strategy to treat cancer. APR-246 is the only mp53 activator that is currently in clinical trials. Our aim was to find potential G245S-mp53 activators. We created atomistic *in silico* models of the mutant protein and virtually screened the ZINC database library using DOCKTITE’s covalent docking protocol. The filtered library was assigned to bind at C124 of G245S-mp53. The ligands were ranked based on consensus scoring. We used both the knowledge-based DSX and Affinity dG empirical scoring functions. We also used ADMET Predictor™ to predict possible toxicities of the compounds. Our results show that compound 2 has the best potential as a G245S-mp53 activator. The minimized structure of the complex composed of Compound 2 and G245S-mp53 protein shows that the compound becomes buried in the protein and its hydrophobic portion forms van der Waals interactions with the hydrophobic core of the protein. *In vitro* testing will be required to validate our predictions and, provided this is successful, further select a subset of the predicted hits for preclinical development. It is hoped that the work reported here opens new avenues for targeting this important cancer biomarker.

## Methods and models

### Ligand library preparation

We screened the ZINC15 database, which originally contains about 13 million compounds[34]. We applied three criteria to filter the compounds and reduce the size of the docked database. Our filtered sub-library contained compounds with molecular weights between 300 and 500 Daltons and had an octanol-water partition coefficient (logP) between -1 and 5. We also limited our search to compounds that were in stock and were categorized as mild to reactive. Our final library size was about 130,000 drug-like compounds. We downloaded the 3D representations of the ligands including their different protomer and tautomers.

### G245S-mp53 preparation

We created the G245S-mp53 models as described in our previous work[25]. Briefly, we used the NMR resolved apo wt-p53 with PDB ID: 2FEJ[35] as well as the X-ray resolved wt p53-DNA complex (PDB ID: 4HJE[36]) as the starting structures for our models. We used PyMol to virtually mutate residue 245 from glycine to serine[37]. We MD-simulated the mutated models for 1 and 1.5 μs, respectively as described in[25].

### RMSD-based clustering

To account for the flexibility of the protein’s binding site using a manageable number of representative protein models, the structure of the last 460 ns of the equilibrated apo G245S-mp53 monomer (from 2FEJ) and the last 1μs of the G245S-mp53 monomer B (from 4HJE) were clustered using the cpptraj utility in Ambertools[27]. Clustering using the average-linkage bottom-up algorithm was based on the root-mean-squared deviation of residues 113–124 and 141–146, constituting the pocket around the C124 site. We used the Davies-Bouldin index (DBI), the pseudo F-statistic (pSF) and the sum of square regression-sum of total sum of square ratio (SSR/SST) clustering metrics to determine the clustering quality and the optimum number of representative clusters[26]. Generally, lower DBI and higher pSF values signal better clustering. The SSR/SST ratio was used following the "elbow criterion" for the choice of the number of clusters[26]. The centroid structures, which have the lowest RMSD to all the other conformations in the cluster, were used to represent the flexibility of the active site during docking.

### Covalent docking using DOCKTITE

We employed the DOCKTITE protocol to virtually screen the filtered sub-library using covalent docking[24]. The first step of the protocol is to screen the molecules for reactive electrophilic warheads. For each compound in the filtered sub-library, the ligands are each attached to the nucleophilic thiol of C124. Additionally, stereoisomers were also created for prochiral compounds. A pharmacophore model of the active site is also automatically generated. The active site was defined by residues 113–124 and 141–146 as well as all atoms within 9 Å from the center of the selected residues. As part of the DOCKTITE protocol, stochastic sampling was used to generate 5,000 possible conformations of the ligands. Docking of the 5,000 conformers was then performed and was guided by the previously generated pharmacophore model. The docked conformers were first evaluated by the empirical Affinity dG scoring function. Based on their scores, the top 100 poses are further refined using the grid minimization method then rescored using the Affinity dG scoring function. For better estimation of the results, the ligands were then cleaved from the nucleophilic side-chains and rescored. Identification of the top hits at this stage was based on consensus scoring. The external knowledge-based scoring function, DSX[38], was used to rank the different poses of a compound based on their similarities to near-native poses. The binding energy of the pose that ranked first with the DSX function was then recalculated with MOE’s Affinity dG empirical scoring function.

### ADMET Predictor™

We used ADMET Predictor™ of SimulationsPlus[39] to predict compound toxicities. ADMET predictor is a machine learning algorithm that calculates various properties and toxicities of compounds and assigns an ADMET risk score to them called ‘ADMET risk’; higher scores indicate less favorable pharmacokinetic and pharmacodynamic properties. We calculated the ligands’ blood brain barrier penetration ‘BBB filter’, their inhibition of the hERG potassium channel of the heart ‘hERG filter’, the likelihood of the compounds to inhibit or be substrates of p-glycoproteins ‘Pgp Inh’ and ‘Pgp substr’, respectively. We also calculated the hepatotoxic potential of the compounds by predicting their effect on serum alanine transaminase ‘Ser ALT’ and aspartate transaminase ‘Ser AST’ liver enzymes.

## Supporting information

S1 Text The PDB file of the minimized G245S-mp53 covalently bound to compound 2. (PDB)
